# Endothelin Receptors Expressed by Immune Cells Are Involved in Modulation of Inflammation and in Fibrosis: Relevance to the Pathogenesis of Systemic Sclerosis

**DOI:** 10.1155/2015/147616

**Published:** 2015-05-18

**Authors:** Tinazzi Elisa, Puccetti Antonio, Patuzzo Giuseppe, Barbieri Alessandro, Argentino Giuseppe, Confente Federico, Dolcino Marzia, Beri Ruggero, Marchi Giacomo, Ottria Andrea, Righetti Daniela, Rampudda Mariaelisa, Lunardi Claudio

**Affiliations:** ^1^Department of Medicine, University of Verona, 37134 Verona, Italy; ^2^G. Gaslini Institute, 16148 Genoa, Italy; ^3^University of Genoa, 16126 Genoa, Italy; ^4^Pederzoli Hospital, Peschiera del Garda, 37019 Verona, Italy

## Abstract

Endothelin-1 (ET-1) plays a pivotal role in vasoconstriction, fibrosis, and inflammation, the key features of systemic sclerosis (SSc). ET-1 receptors (ET_A_ and ET_B_) are expressed on endothelial cells, smooth muscle cells, and fibroblasts, but their presence on immune cells has not been deeply investigated so far. Endothelin receptors antagonists such as bosentan have beneficial effects on vasoconstriction and fibrosis, but less is known about their potential anti-inflammatory effects. We studied the expression of ET-1 receptors on immune cells (T and B lymphocytes, monocytes, and neutrophils) and the link between ET-1 and inflammation in patients with SSc. We show here that ET-1 exerts a proinflammatory effect in CD4+ T cells, since it induces an increased IFN-*γ* production; preincubation with antagonists of both receptors reduces IFN-*γ* production. Moreover, following ET-1 stimulation, neutrophils produce proinflammatory mediators, thus amplifying the effects of activated CD4+ T cells. Our data indicate that ET-1 system is involved in the pathogenesis of inflammation and fibrosis typical of SSc, through the activation of T lymphocytes and neutrophils and the consequent release of proinflammatory and profibrotic cytokines. These findings suggest that dual ET-1 receptors antagonist therapy, besides its effect on vasculopathy, has a profound impact on the immune system favouring antiinflammatory and antifibrogenic effects.

## 1. Introduction

Systemic sclerosis (SSc) is an autoimmune disease that involves the connective tissue of skin and internal organs with a remarkable heterogeneity in the disease course and affected organs, resulting in high morbidity and mortality. The disease is characterized by vascular dysfunction and injury and by overproduction and accumulation of collagen and other extracellular matrix proteins, resulting in the thickening of the skin and fibrosis of the affected organs [1, 2]. The pathogenetic mechanisms involve three interactive components represented by severe and diffuse endothelial cell damage, immune system dysfunction, and fibroblasts activation.

Endothelin-1 (ET-1) has been described to play a role in fibrosis, angiogenesis, and inflammation, all major features of SSc [3, 4]. Indeed ET-1 level is elevated in the serum and tissues of SSc patients, especially in diffuse SSc patients, and serum levels have been shown to correlate with the extent of vascular damage and cutaneous fibrosis [3–6].

ET-1 is the major isoform of three endothelin isoforms and is a soluble mediator that exerts a potent vasoconstrictor effect [7]. ET-1 has been firstly described in endothelial cells and in vascular smooth muscle cells, where hypoxia, cold exposure, low shear stress, angiotensin II, cytokines, and growth factors may facilitate its production [7, 8]. Many cells can produce ET-1 including fibroblasts and myofibroblasts, mast cells, monocytes/macrophages, polymorphonuclear leukocytes, and dendritic cells [9–14]. Transforming growth factor- (TGF-) *β* and ET-1 itself, with an autocrine loop, are able to induce ET-1 production in fibroblasts and myofibroblasts [9, 15].

There are at least three ET-1 receptors: ET_A_, ET_B_, and ET_C_ [8, 16, 17]; however, the function of ET_C_ is poorly known; ET_A_ and ET_B_ are expressed on the majority of cells that actively contribute to SSc pathogenesis, such as fibroblasts, myofibroblasts, vascular smooth muscle cells, and platelets, while endothelial cells selectively express ET_B_ [6–8, 16]. Upon binding receptors on vascular smooth muscle cells, ET-1 is able to induce vasoconstriction, cell growth, and proliferation, leading to lumen narrowing at arterial and arteriolar level [18–20]. Moreover, ET-1 facilitates fibroblasts transdifferentiation into myofibroblasts and induces the production of both collagen and ET-1 probably through an autocrine mechanism [9, 15, 21–26].

There is increasing evidence that ET-1 may play a pivotal role in inflammation in several human diseases including chronic renal disease, asthma [27–30], and sepsis (reviewed in [44]); however, the mechanisms by which ET-1 induces the activation of the innate and adaptive immune systems have not been fully elucidated so far. Saleh and Pollock suggested that ET-1 can directly activate neutrophils and can induce the production of chemoattractant factors, such as monocyte chemoattractant factor-1 (MCP-1), and the synthesis of cell adhesion molecules, such as soluble intercellular adhesion molecule-1 (ICAM-1) [27]. Moreover ET-1 seems to be associated with the activation of transcription factors such as NF-*κ*B and the production of proinflammatory cytokines including tumor necrosis factor-alpha (TNF-*α*), interleukin- (IL-) 1, and IL-6 [31]. Little is known on ET-1 receptors expression on immune cells with the exception of a few data on cells of the innate immune system, such as dendritic cells and monocytes [10–14, 32]. The expression of ET-1 receptors on adaptive immune effectors cells (T and B lymphocytes) has not been investigated so far and therefore little is known on the possible role of ET-1 as a mediator of inflammatory responses.

In the last decade, orally active ET-1 receptor antagonists (ERAs) were developed and approved for clinical use. Two orally active ERAs are currently approved, the dual receptor antagonist, bosentan, and the selective ET_A_ receptor antagonist, ambrisentan [33–35]. Both ERAs are used in the treatment of pulmonary arterial hypertension (PAH) whereas only bosentan has been shown to be effective in the prevention of new scleroderma-related digital ulcers (DUs) [36]. In addition to the effects on vasculature and fibrosis [36–38], it has been recently reported that ET-1 blockade using bosentan may also have some anti-inflammatory effects ([39], reviewed in [44]). In particular, bosentan seems to be able to suppress the ET-1-induced production of TNF-*α* and other proinflammatory mediators by monocytes* in vitro* [11].* In vivo*, bosentan significantly reduces IL-6, ICAM-1, and pro-brain natriuretic peptide (pro-BNP) serum levels in patients with PAH [40] and leads to the normalization of soluble adhesion molecules in SSc-associated PAH [40, 41].

Inflammation is deeply involved both in the early phase of SSc pathogenesis and in the progression of vascular damage and fibrosis. Therefore, we aimed at investigating the role of ET-1 as possible mediator of inflammatory damage in SSc. Since immune effectors cells, such as T and B lymphocytes, monocytes, and neutrophils, are important players of inflammation in SSc, we aimed at clarifying the possible role played by ET-1 receptors in immune cells activation.

In this paper, we studied the presence of ET-1 receptors on T and B lymphocytes, monocytes, and neutrophils by FACS analysis. We also analysed the effects of ET-1 receptors engagement in order to verify the proinflammatory activity of ET-1 and the potential anti-inflammatory effects of ERAs.

## 2. Materials and Methods

### 2.1. Patients and Controls

We studied a cohort of 41 patients (5 males and 36 females, mean age: 57 ± 14 years) affected by SSc, attending the Unit of Autoimmunity Diseases at the University Hospital of Verona, Italy. SSc diagnosis was performed in accordance with the American College of Rheumatology/European League against Rheumatism classification criteria for systemic sclerosis [42, 43].

Patients were classified according to the following clinical features: limited (lSSc) or diffuse (dSSc) cutaneous form of SSc (32 patients with lSSc and 9 with dSSc) and presence or absence of ischemic digital ulcers, PAH, and interstitial lung disease (ILD). Ten patients were on bosentan therapy because of digital ulcers or PAH. Twenty age and sex matched healthy subjects were used as control group.

Blood samples (20 mL) were collected in heparinized Falcon tubes (Becton Dickinson, NJ, USA) from both patients and control subjects. A written informed consent was obtained from all the participants to the study and the study was approved by the local ethical committee. All clinical investigations have been conducted according to the principles expressed in the Helsinki declaration.

### 2.2. Isolation of Peripheral Blood Mononuclear Cells and Flow-Cytometry

Blood samples obtained from patients and controls were diluted with 20 mL of phosphate buffered saline (PBS) solution. Mononuclear cells isolated by density gradient centrifugation using lymphoprep Ficoll-Isopaque (Axis-Shield, Oslo, Norway) were washed twice with PBS and suspended in tubes containing 1 million cells for flow-cytometry (FACS) analysis. Analysis of monocytes and lymphocytes was carried out in different tubes; cells used for monocytes staining were preincubated with mouse serum (DAKO, Glostrup, Denmark) for 10 minutes at room temperature. Each sample was incubated for 1 hour at 4°C with eitherrabbit polyclonal anti-ET_A_ (Acris Antibodies GmbH, Herford, Germany) or sheep polyclonal anti-ET_B_ (Lifespan Biosciences, Seattle, WA, USA) antibodies. Phycoerythrin- (PE-) conjugated goat anti-rabbit IgG monoclonal (0.25 mg/mL) was used as a secondary antibody for ET_A_ (R&D Systems, Minneapolis, MN, USA) and PE-conjugated donkey anti-sheep IgG monoclonal (0.2 mg/mL) was used as a secondary antibody for ET_B_ (R&D Systems) and incubated for 30 minutes at 4°C. Samples were also stained for 20 minutes at room temperature in a dark room with allophycocyanin- (APC-) conjugated anti-CD3 or anti-CD14 or anti-CD19 antibodies (BD Biosciences, San Jose, CA, USA). After labeling, samples were acquired in a FACSCanto cytometer (Becton Dickinson). The sensitivity of fluorescence detectors was set and monitored using Calibrite Beads (Becton Dickinson) according to the manufacturer's recommendations; 20.000 CD3+, CD14+, or CD19 cells per sample were, respectively, acquired in live gating. FlowJo 8.8.2 software (Tree Star, Ashland, OR) was used to analyze the data. Expression of ET_A_ or ET_B_ was calculated as the difference between mean fluorescence intensity (MFI) of cells stained with primary plus secondary antibodies and MFI of their negative control (cells stained with secondary antibodies): ΔMFI.

### 2.3. T Cells Stimulation

In order to assess receptors expression on activated CD4+ and CD8+ T cells, PBMC isolated from 4 patients and 4 controls were stimulated for 24 hours with anti-CD3/CD28 antibodies coated microbeads-Dynabeads Human T-Activator (Dynal, Oslo, Norway), according to the manufacturer's recommendations. Cells were cultured in RPMI-1640-GlutaMAX-I, supplemented with 10% fetal calf serum (FCS), 100 U/mL penicillin, and 100 microg/mL streptomycin (all purchased from Life Technologies, Carlsbad, CA). In order to identify CD4+ and CD8+ T lymphocytes, we incubated cells with a mixture of the following antibodies: PerCp-conjugated anti-CD3, APC-H7-conjugated anti-CD4, and APC-conjugated anti-CD8 antibodies. Activated cells were detected by incubating cells with FITC-conjugated anti-CD25 antibodies; all reagents were purchased from BD Biosciences. Cells were previously stained with anti-ET_A_ and anti-ET_B_ primary and secondary antibodies as previously described and samples were acquired on a FACSCanto cytometer FlowJo 8.8.2 software was used to analyse data. The variation in receptors surface exposure was expressed as the difference between activated cells MFI and unstimulated cells MFI (ΔΔMFI).

### 2.4. Isolation of T CD4+ Cells

Mononuclear cells from healthy donors buffy coats were isolated by density gradient centrifugation using lymphoprep Ficoll-Isopaque. CD4+ T cells were obtained through negative selection using CD4+ T Cell Isolation Kit II (Miltenyi Biotec) and MidiMACS Starting Kit, including MACS LD column and MACS Separator (Miltenyi Biotec), following manufacturer's instructions.

### 2.5. RNA Extraction and RT-PCR from CD4+ T Cells

Total RNA was extracted from CD4+ T cells using TRIzol Reagent (Gibco BRL, Billings, MT, USA) following the manufacturer's protocol. RNA was previously treated with DNAse I (Invitrogen).

First-strand cDNA was carried out using the Super Script III System (Invitrogen, Carlsbad, CA, USA), with random hexamers, according to the manufacturer's recommendations. Fibroblasts cDNA was used as positive control for the detection of ET_A_- and ET_B_-coding mRNA.

CD4+ T cells and fibroblasts cDNA were amplified with ET_A_ and ET_B_ specific primers:

 ET_A_ forward 5′-ATGCACAACTATTGCCCACA-3′, ET_A_ reverse 5′-GGACAGGATCCAGATGGAGA-3′;
 ET_B_ forward 5′-GCACATCGTCATTGACATCC-3′, ET_B_ reverse 5′-CAGAGGGCAAAGACAAGGAC-3′ (Sigma-Aldrich, Saint Louis, MO, USA).

Vimentin was used as PCR reaction-control. Amplification was performed using the AmpliTaq Gold PCR MasterMix system (Applied Biosystems, Foster City, CA, USA). cDNA was amplified using the primers specific for ET_A_ and ET_B_ receptors and for vimentin using the GeneAmp PCR System 9700 thermal cycler (Applied Biosystems) and the amplification reaction was carried out as follows: 10 minutes at 95°C followed by 40 cycles of denaturation (45 seconds at 94°C), annealing (30 seconds at 53°C for ET_A_ and 55°C for ET_B_ and for vimentin), and extension (1 minute at 72°C and 7 minutes at 72°C to stop reaction). Amplicons (length: 447 bp for ET_A_, 558 bp for ET_B_, and 266 bp for vimentin) were run on agarose gel (1.5%) and revealed using VersaDoc video documentation system (Bio Rad, Hercules, CA, USA).

### 2.6. Evaluation of Cytokine Secretion by CD4+ T Lymphocytes following ET_A_ and ET_B_ Stimulation

In order to study the cytokine production in response to ET_A_ and ET_B_ stimulation by ET-1 in CD4+ T cells, we seeded CD4+ cells in microplates: 1 million CD4+ T cells per well were seeded in 24-well plates and different conditions were carried out in duplicate. Cells were incubated (a) without ET-1 and receptors antagonists (control sample); (b) with ET-1 alone; (c) with ET_A_ antagonist (BQ123) and ET-1; (d) with ET_B_ antagonist (BQ788) and ET-1; (e) with BQ123 plus BQ788 and ET-1. Cells were incubated with BQ123 and BQ788 at the concentration of 10^−6 ^M for 45 minutes and with ET-1 at concentration of 10^−7 ^M for 24 hours. All reagents were purchased from Sigma-Aldrich.

We then measured interferon- (IFN-) *γ*, IL-4, and IL-17 concentrations in cell culture supernatants by enzyme-linked immunosorbent assay (ELISA) (Quantikine Human IFN-*γ* Immunoassay, Quantikine Human IL-4 Immunoassay, and Quantikine Human IL-17 Immunoassay, resp., obtained from R&D Systems), following the manufacturer's instructions. Sunrise absorbance reader for microplates (Tecan, Salzburg, Austria) was used to determine optical density for each sample.

### 2.7. Isolation of Neutrophils, Flow-Cytometry, and RT-PCR

We isolated neutrophils from healthy donors buffy coat in order to study surface expression of ET_A_ and ET_B_ by flow-cytometry and the transcripts for ET_A_ and ET_B_ by RT-PCR. Highly purified granulocytes (neutrophils > 96.5%) were isolated and prepared under endotoxin-free conditions using lymphoprep Ficoll-Isopaque. Neutrophils were further enriched by positively removing all contaminating cells with mAb against CD3, CD56, CD19, CD36, CD49d, and Gly-A using a custom-made Easy-Sep kit (StemCell Technologies, Vancouver, BC, Canada) to reach more than 99,7% purity. One million neutrophils were suspended in tubes for FACS analysis. Staining for ET_A_- and ET_B_ was carried out as already described. RNA extraction and RT-PCR were performed as previously described.

### 2.8. Analysis of Cytokines and MMP-9 in the Supernatants of Neutrophils Stimulated with ET-1

Neutrophils were seeded in microplates and incubated with or without 100 ng/mL Ultrapure* E. coli* lipopolysaccharide (LPS) (Invivogen, San Diego, CA) and with or without ET-1. Therefore we used 4 different conditions: (a) neutrophils without any stimulus as negative control, (b) neutrophils incubated with ET-1, (c) neutrophils incubated with LPS alone, (d) and neutrophils incubated with both ET-1 and LPS. Cells were cultured in RPMI-1640-GlutaMAX-I, supplemented with 10% fetal calf serum (FCS), 100 U/mL penicillin, and 100 microg/mL streptomycin. Interleukin-8, TNF-*α*, vascular endothelial growth factor (VEGF), IFN-*γ*, IL-17, and matrix metallopeptidase 9 (MMP-9) released in the supernatants of cultured neutrophils were assessed by ELISA at two different time points (3 and 10 hours). Quantikine Human Immunoassay for the selected molecules was purchased from R&D Systems. Sunrise absorbance reader for microplates was used to determine optical density for each sample.

### 2.9. Statistical Analysis

All the calculations were performed with SPSS 21.0 statistical package (SPSS Inc., Chicago, IL, USA). All the results are expressed as ΔMFI mean ± standard deviation. Quantitative data were assessed using Student's *t*-test. Correlations between ET-A and ET-B cell surface expression and clinical features were assessed with nonparametric test and multivariate analysis. A value of *P* < 0.05 was considered statistically significant.

## 3. Results

### 3.1. ET-1 Receptors Expressed by Immune Effector Cells

**Figure 1:**
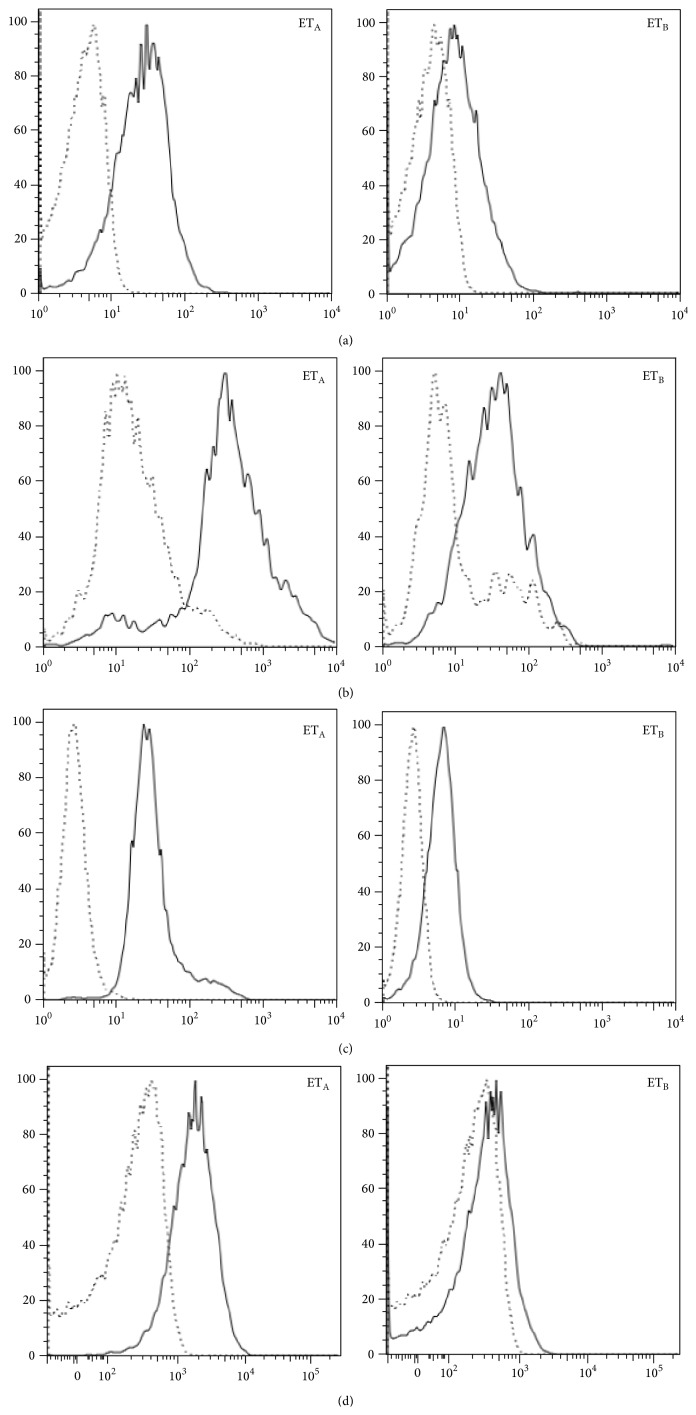
ET_A_ and ET_B_ expression by cells obtained from SSc patients. The quantification of receptors expression by T (a) and B lymphocytes (b), monocytes (c), and neutrophils (d) is represented by the difference of fluorescence intensity between the sample (continuous line) and its negative control (dotted line). The profile of one of 41 SSc patients is shown. All the other patients had a similar behaviour.

**Figure 2:**
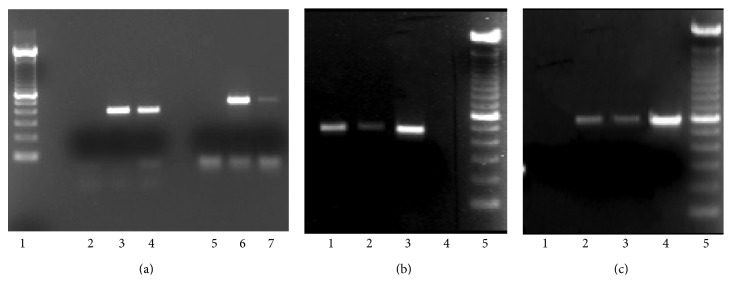
ET_A_ and ET_B_ transcripts amplified by RT-PCR in fibroblasts, CD4+ T lymphocytes, activated CD4+ T cells, and neutrophils. ET_A_ corresponds to a molecular weight of 446 bp and ET_B_ to a molecular weight of 558 bp. (a) ET_A_ and ET_B_ transcripts amplified by RT-PCR in fibroblasts and neutrophils. Lane 1: molecular weight ladder; lane 2: negative control; lane 3: fibroblasts (ET_A_); lane 4: neutrophils (ET_A_), lane 5: negative control; lane 6: fibroblasts (ET_B_); lane 7: neutrophils (ET_B_). (b) ET_A_ transcripts amplified by RT-PCR in T lymphocytes, activated T cells, and fibroblasts. Lane 1: T lymphocytes; lane 2: activated T cells; lane 3: fibroblasts; lane 4: negative control, lane 5: molecular weight ladder. (c) ET_B_ transcripts amplified by RT-PCR in T lymphocytes, activated T lymphocytes, and fibroblasts. Lane 1: negative control; lane 2: T lymphocytes; lane 3: activated T cells; lane 4: fibroblasts; lane 5: molecular weight ladder.

T and B lymphocytes as well as monocytes and neutrophils express ET_A_ and ET_B_ on their surface, using FACS analysis; the data were obtained as a difference of mean fluorescence intensity between samples incubated with primary and secondary antibodies and their negative controls incubated with secondary antibody alone (Figure 1). In addition some of the data were validated by reverse transcription-PCR in CD4+ T cells and neutrophils (Figure 2).

In both patients and controls, T lymphocytes and monocytes showed a higher surface expression of ET_A_ (patients: ΔMFI = 100.61 ± 45.21 and 212.24 ± 64.27, resp.; controls: ΔMFI = 110.45 ± 35.89 and 188.4 ± 35.61, resp.) when compared to ET_B_ (patients: 46.85 ± 29.78 and 91.14 ± 27.44, resp.; controls: ΔMFI = 49.23 ± 29.16 and 98.74 ± 54.66, resp.) (*P* < 0.001) (Table 1).

These data indicate that surface ET-1 receptors distribution on T cells and monocytes of SSc patients is similar to the one observed in healthy donors.

Patients affected by dSSc showed a lower ET_B_ surface expression on T cells when compared to patients affected by lSSc (28.6 ± 17.9 versus 51.9 ± 31.1) (*P* < 0.01); a similar pattern of surface expression was observed on monocytes (74.4 ± 29.6 versus 97.2 ± 24.5) (*P* < 0.05). No significant difference in ET_A_ expression by T lymphocytes and monocytes was observed in the diffuse or limited form of disease (94.8 ± 48.2 versus 99.1 ± 42.1 and 251.2 ± 116.3 versus 199.3 ± 34.5, resp.).

ET_A_ and ET_B_ surface expression were not modified by bosentan treatment, both on T cells (ET_A_: 97.9 ± 52 versus 102.6 ± 44.3; ET_B_: 47.9 ± 17.7 versus 47.6 ± 32.3) and on monocytes (ET_A_: 240.7 ± 130.6 versus 205.1 ± 34.9; ET_B_: 89.6 ± 22.9 versus 93.1 ± 27.7), suggesting that bosentan therapy does not induce an increased ET-1 receptors expression.

ET_A_ and ET_B_ surface expression on T cells and monocytes did not correlate with the presence or absence of DUs (T cells: 121.4 ± 69 versus 98.8 ± 41.6 and 40.8 ± 20.1 versus 48.6 ± 31, resp.; monocytes: ET_A_: 221 ± 4.3 versus 211.3 ± 69.6; ET_B_: 80.4 ± 25.3 versus 93.6 ± 27.5) (Table 2).

Patients with PAH had a lower ET_B_ surface expression on monocytes when compared to patients without PAH, although the difference was not statistically significant (77.2 ± 23.4 versus 96.9 ± 27.3); this difference was significant when considering patients with the limited subset of the disease (77.6 ± 17.6 versus 102.3 ± 24.4; *P* < 0.05) (Table 2).

Furthermore, ET_A_ expression was lower on T cells of lSSc patients with ILD when compared to T cells of patients without ILD (77.8 ± 34.2 versus 111.6 ± 43.9; *P* < 0.05) (Table 2).

ET_A_ and ET_B_ expression on B lymphocytes were similar in patients and healthy donors (Table 1). In SSc patients ET_A_ surface expression was higher than ET_B_ and was not influenced by the treatment with bosentan (ET_A_: 281.33 ± 43.47 and ET_B_: 161.33 ± 43.97; *P* < 0.05 versus ET_A_: 270.00 ± 28.16 and ET_B_: 171.33 ± 35.47; *P* < 0.05). ET_A_ and ET_B_ surface expression were similar in the diffuse or limited form of disease and were not influenced by the presence of PAH, ILD, and DUs.

Neutrophils presented the same pattern of expression of ET-1 receptors in SSc patients and control subjects (Table 1).

### 3.2. Quantification of ET_A_ and ET_B_ Expression on Activated T CD4+ and CD8+ Cells

**Figure 3:**
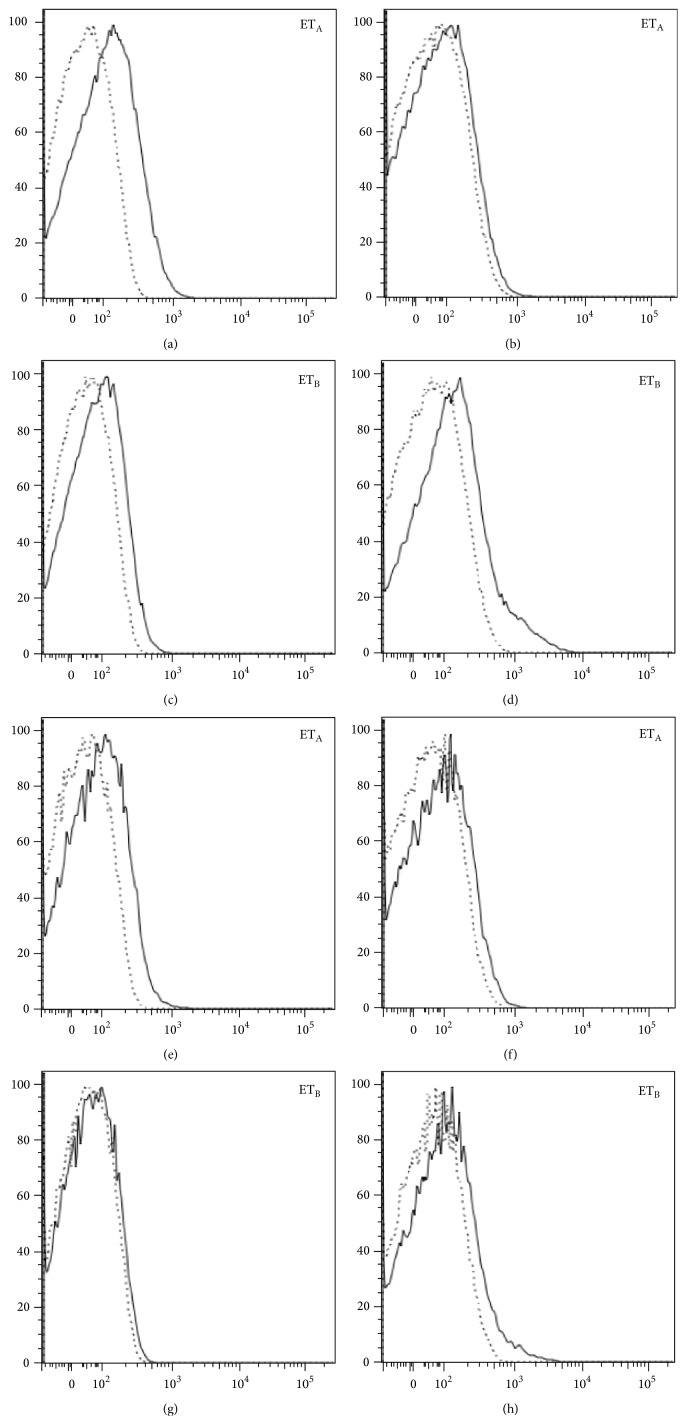
Change in ET_A_ and ET_B_ expression on activated T CD4+ and CD8+ cells. The stimulation of cells, performed with microbeads coated by anti-CD3/CD28 antibodies, leads to a reduction of ET_A_ and an increase of ET_B_ expression, both in CD4+ (a-b; c-d) and CD8+ cells (e-f; g-h), respectively. The profile of one of ten similar experiments is shown.

As already shown on the entire T cell population, both CD4+ and CD8+ T cell subsets isolated from SSc patients and control healthy donors express ET_A_ and ET_B_ on their surface. A higher ET_A_ expression on resting CD4+ and CD8+ T cells was also confirmed. Upon activation, we found a decreased expression of ET_A_ and an increased expression of ET_B_ (Figure 3).

### 3.3. Cytokines Production by CD4+ T Cells following ET_A_ and ET_B_ Stimulation by ET-1

We tested the levels of INF-*γ*, IL-4, and IL-17 in the supernatants of CD4+ T cells obtained from SSc patients and controls. Cells were either incubated without any stimulus or incubated with ET-1 in the presence or absence of ET-1 receptors blockade. After 24 hours of incubation with ET-1, INF-*γ* concentration was 9.5 times higher than in the supernatants of cells cultured without ET-1 (7.6 pg/mL versus 0.8 pg/mL; *P* < 0.05). Following selective ET_A_ or ET_B_ blockade before ET-1 stimulation, INF-*γ* levels decreased to 1.2 and 1.6 pg/mL, respectively. Remarkably, the simultaneous dual ET-1 receptors blockade, which mimics* in vitro* the effects of bosentan treatment, caused a marked reduction of INF-*γ* concentrations in the cells culture supernatants. Interleukin-4 levels did not change in the supernatant of cells exposed to ET-1 for 24 hours. In the presence of selective inhibition of ET_A_, IL-4 levels increased in a more significant manner than in the presence of ET_B_ blockade (711.42 ± 102.2 pg/mL and 694.47 ± 99.8 pg/mL, resp., versus 60.8 ± 80.2; *P* = 0.018 and *P* < 0.01). Also, the double receptors blockade induced the production of IL-4 (682 ± 100.6 pg/mL versus 60.8 ± 80.2 pg/mL; *P* = 0.02). Remarkably, we observed a slight increase in IL-17 level following the incubation with ET-1. However, this effect was not modified by partial or complete ET-1 receptor blockade (Table 2).

### 3.4. Molecules Released by Neutrophils after ET_A_ and ET_B_ Stimulation by ET-1

Levels of MMP-9, IL-8, TNF-*α*, VEGF, IFN-*γ*, and IL-17 were evaluated in the medium of neutrophils isolated from 4 healthy donors incubated with ET-1 or LPS or with ET-1 and LPS for 1, 3, or 10 hours (Table 3).

A 1-hour incubation with ET-1 induced a marked increase of MMP-9 (165.1 ng/mL versus 46.4 ng/mL; *P* < 0.05), whereas, at the same time point, we did not observe significant changes in the production of the other soluble molecules analysed, as shown in Table 4. After 3 hours of incubation with both ET-1 and LPS, neutrophils released a higher amount of IL-8 when compared to the concentration detected following the incubation with ET-1 alone or LPS alone (67.7 versus 7.7 versus 59.2 pg/mL, resp.), whereas the levels of the other soluble mediators remained unchanged.

The concentration of TNF-*α* released in the supernatants following a 10-hour incubation with ET-1 was higher than the levels detected in the medium of cells stimulated with ET-1 and LPS or LPS alone (23.4 pg/mL versus 2.1 pg/mL versus undetectable level; *P* < 0.05). At the same time point, incubation with ET-1 alone induced a higher production of IL-17 compared to the stimulation with both ET-1 and LPS or LPS alone (3.5 pg/mL versus undetectable level; *P* < 0.05). Finally, the simultaneous stimulation with ET-1 and LPS induced a higher secretion of IL-8 and MMP-9 in the supernatant (134.6 pg/mL and 220.7 ng/mL, resp.) when compared to the concentration reached with ET-1 (16.6 pg/mL and 63.7 ng/mL, resp.) or LPS alone (93.8 pg/mL and 205.3 ng/mL, resp.).

Taken together, these data indicate that ET-1 is able to induce neutrophils to release proinflammatory mediators.

## 4. Discussion

In the present study, we aimed firstly at analysing the cellular surface distribution of ET-1 receptors in the different immune cell subsets and secondly at dissecting the mechanisms by which the ET-1 signalling network may participate in the inflammatory responses in SSc.

ET-1 is a potent vasoconstrictor which plays a fundamental role in key pathogenetic aspects of SSc such as vascular damage and fibrosis and treatment with ERAs exerts beneficial effects on vasculopathy [36, 37]. More recently, amelioration of inflammatory parameters during ERA treatment has been reported (reviewed in [44]), thus implying an important role for ET-1 in inflammation, another important aspect of SSc pathology. Indeed inflammation plays a pivotal role in early SSc [45]; however, it may influence also different phases of SSc.

The presence of ET-1 receptors on dendritic cells and polymorphonuclear cells has been already reported [6–8, 16], often with conflicting results, whereas very little is known on ET_A_ and ET_B_ expression on T and B lymphocytes. Moreover, a proinflammatory role for ET_B_ expressed by monocytes/macrophages has been hypothesized on the basis of increased production of inflammatory mediators (TNF-*α*, prostaglandin E2, and IL-1*β*) upon ET-1 stimulation [11, 44].

We show here that all the immune cells studied (B and T lymphocytes, monocytes, and neutrophils) express ET-1 receptors both in normal subjects and in SSc patients with a difference in the relative expression of either ET_A_ or ET_B_ in the different cell types analysed. In particular, B lymphocytes and neutrophils show the same pattern of expression in healthy controls and in SSc patients, without any significant difference related to the clinical features of the disease. T lymphocytes and monocytes express a higher ET_A_ expression than ET_B_ on both subsets. Since ET-1 serum levels are higher in dSSc than lSSc patients and they correlate with the extent of vascular damage and cutaneous fibrosis, we may hypothesize that at least part of ET-1 profibrotic effects is preferentially mediated by the engagement of ET_A_ [3, 16].

Interestingly, we noticed that, in lSSc patients, a lower ET_B_ expression on monocytes correlates with the presence of PAH and a lower ET_A_ expression on T cells correlates with ILD. We can therefore hypothesize that a different pattern of receptor expression on immune cells is associated with a different functional activity that may contribute to the development of PAH or ILD.

A recent multicenter, placebo-controlled trial investigating new drug therapies for idiopathic pulmonary fibrosis compared the effects of ambrisentan, a selective ET_A_ antagonist, to placebo on disease progression. The study showed that the treatment was associated with an accelerated decline in pulmonary function tests, increased hospitalizations, and higher mortality [46]. Since a selective ET_A_ inhibition, such as the one obtained with ambrisentan, seems to accelerate pulmonary disease, we may suggest that an imbalanced expression of ET-1 receptors with a diminished expression of ET_A_ on immune cells may predispose SSc patients to develop ILD possibly through an increased ET_B_-mediated stimulation on T cells. The results of this study are in accordance with our findings of a lower ET_A_ expression on T cells of SSc patients with ILD. However, the precise role played by ET_A_ stimulation and inhibition in the progression of pulmonary fibrosis remains unclear.

We next evaluated whether the presence of an inflammatory microenvironment could influence the relative expression and/or distribution of ET-1 receptors and, to this aim, we stimulated T cells with anti-CD3/CD28 antibody-coated microbeads. Stimulation resulted in reduced expression of ET_A_ and increased expression of ET_B_ on CD4+ and CD8+ T cells, thus suggesting that these cells, once activated, modulate receptor surface expression by overexpressing ET_B_ and downregulating ET_A_. These results support the hypothesis that ET_B_ signalling plays a major role in inflammation and, as a consequence, that dual ET-1 receptors blockade may represent a more suitable therapeutic strategy in SSc. We have then investigated the functional effects of ET-1 stimulation on CD4+ T cells and found that ET-1 is able to induce a proinflammatory response since the engagement of both ET_A_ and ET_B_ induced an IFN-*γ* secretion 9.5 times higher than the one observed in basal condition. IFN-*γ* production was markedly reduced following dual receptor blockade, a situation which resembles the effect of bosentan treatment. These findings suggest the need of ET_A_-ET_B_ receptors cooperation to obtain an inflammatory response in CD4+ lymphocytes. These results are in agreement with the observation that simultaneous blockade of both ET_A_ and ET_B_ in scleroderma fibroblasts is necessary in order to suppress collagen production [24]. In addition, ET-1 stimulation of CD4+ T cells leads to a mild increase of IL-17 concentration in cell supernatants, suggesting again an important role for ET-1 in inducing the production of proinflammatory cytokines. Finally, the receptors blocking induces the production of the anti-inflammatory cytokine IL-4.

Finally, it is interesting to note that neutrophils activated with LPS are able to increase the production of proinflammatory molecules after stimulation with ET-1, thus giving further support to the proinflammatory effects of ET-1 also on cells of innate immunity.

All together, these data indicate that ET-1 behaves also as a proinflammatory molecule through a synergistic action on ET_A_ and ET_B_. Therefore, a dual receptor blockade strategy is likely to better control inflammation and fibrosis than a selective receptor blockade. In conclusion, our results, besides generating useful insight in the understanding of ET-1 effects on immune cells in healthy donors and in SSc patients, provide a rationale for the use of dual receptor antagonist in the early stages of SSc, when inflammation is prominent.

## Figures and Tables

**Table 1:** ET_A_ and ET_B_ expression on T and B cells, monocytes, and neutrophils in healthy controls and SSc patients.

ET-1 receptors	T lymphocytes		B lymphocytes		Monocytes		Neutrophils	
	ET_A_	ET_B_	ET_A_	ET_B_	ET_A_	ET_B_	ET_A_	ET_B_
Healthy controls (*n* = 20)	110.45 ± 35.89	49.23 ± 29.16	269.75 ± 37.14	150.75 ± 26.42	188.4 ± 35.61	98.74 ± 54.66	191.65 ± 42.61	92.54 ± 50.89
SSc patients (*n* = 41)	100.61 ± 45.21	46.85 ± 29.78	253.5 ± 40.54	161.33 ± 43.97	212.24 ± 64.27	91.14 ± 29.16	205.74 ± 59.67	88.34 ± 36.78

Data are expressed as mean ± standard deviation of ΔMFI determined by FACS analysis.

**Table 2:** ET_A_ and ET_B_ expression on T lymphocytes and monocytes in relation to the clinical features of the disease, such as cutaneous form and presence or absence of PAH, ILD, and DUs.

	T lymphocytes		Monocytes	
	ET_A_	ET_B_	ET_A_	ET_B_
lSSc (*n* = 32)/dSSc (*n* = 9)	99.1 ± 42.1/94.8 ± 48.2	51.9 ± 31.1/28.6 ± 17.9 (*P* < 0.01)	199.3 ± 34.5/251.2 ± 16.3	97.2 ± 24.5/74.4 ± 29.6 (*P* < 0.05)
PAH presence/absence	102.6 ± 45.3/104.7 ± 40.9	47.2 ± 26.8/44.9 ± 30.1	202.7 ± 31.4/200.8 ± 30.9	77.2 ± 23.4/96.9 ± 27.3
ILD presence/absence	111.6 ± 43.9/77.8 ± 34.2 (*P* < 0.05)	44.8 ± 27.3/45.3 ± 21.6	199.8 ± 56.5/211.2 ± 47.3	90.6 ± 26.5/89.7 ± 31.7
DUs presence/absence	121.4 ± 69/98.8 ± 41.6	40.8 ± 20.1/48.6 ± 31	221 ± 4.3/196.2 ± 69.6	80.4 ± 25.3/93.6 ± 27.5

**Table 3:** Detection of cytokines in the supernatants of CD4+ T lymphocytes after 24 hours of incubation with ET-1 alone or with selective or dual receptors blockade. One million cells were incubated in each cell culture condition.

	Control cells	Cells with ET-1	Cells with ET-1 and ET_A_ antagonist	Cells with ET-1 and ET_B_ antagonist	Cells with ET-1 and dual receptor blockade
INF-*γ* (pg/mL)	0.8 ± 0.2	7.6 ± 0.2	1.2 ± 0.45	1.6 ± 0.6	0 ± 0.1
IL-4 (pg/mL)	78.1 ± 74.3	60.8 ± 80.2	711.42 ± 102.2	694.47 ± 99.8	682 ± 100.6
IL-17 (pg/mL)	28.7 ± 10.2	36.1 ± 11.1	37.2 ± 9.8	35.6 ± 12.3	36.6 ± 10.8

**Table 4:** Molecules detected in neutrophils supernatants after 1, 3, or 10 hours of incubation with either no stimulus or ET-1, LPS, and ET-1 plus LPS, respectively.

	No stimulus			ET-1			LPS			ET-1 + LPS		
Time of incubation	1	3	10	1	3	10	1	3	10	1	3	10
IL8 (pg/mL)	5.38	6.92	16.92	7.69	7.69	16.92	31.54	59.23	93.85	18.46	67.69	134.6
TNF*α* (pg/mL)	0	0	0	0	0	23.4	0	0	0	0.7	0	2.1
MMP9 (ng/mL)	46.4	67.8	58.8	165.1	64.7	63.3	131.3	203.8	205.3	102.9	205.6	220.7
VEGF (pg/mL)	18.58	20.42	28.75	16.25	22.1	28.7	33.75	52.92	57.92	32.08	54.58	56.25
INF*γ* (pg/mL)	0.28	0	0	1.94	0	0.28	14.17	0	0	0	0	0
IL17 (pg/mL)	0	0	0	0	0	3.5	0	0	0	0	0	0
